# From eco-affective narrowing to agency: acceptance, externalization, and efficacy in climate-related cognition

**DOI:** 10.3389/fcogn.2026.1927324

**Published:** 2026-09-29

**Authors:** Paolo Raile

**Affiliations:** Faculty for Psychotherapy Science, Sigmund Freud University, Vienna, Austria

**Keywords:** acceptance, agency, climate action, climate emotions, collective efficacy, eco-affective cognition, eco-anxiety, externalization

## Abstract

Climate change is increasingly recognized not only as an environmental hazard but also as a condition that reorganizes perception, attention, future-oriented worry, moral appraisal, efficacy judgments, and decision-making. This conceptual analysis reframes eco-anxiety as a paradigmatic form of eco-affective cognition: a dynamic coupling of ecological threat salience, future simulation, responsibility appraisal, and efficacy appraisal under environmental uncertainty. The article develops the Acceptance–Externalization–Agency (AEA) model as a recursive, hypothesis-generating process framework specifying conditions under which eco-affective distress may become more or less action-enabling. The model is not proposed as a linear stage model, a validated clinical protocol, or a therapeutic promise that anxiety can be converted into action. Acceptance reduces secondary struggle against legitimate climate emotions; representational externalization converts diffuse, sometimes preverbal or embodied distress into observable, communicable, and revisable form; responsibility contextualization reduces over-individualized self-blame without dissolving personal, collective, or institutional responsibility; and agency emerges when action experience updates self-efficacy, collective efficacy, hope, and social identity. Selected therapeutic traditions are used not as competing treatments or efficacy claims, but as conceptual exemplars of attentional reorientation, symbolic distancing, embodied perspective shifting, and efficacy-linked action selection. The model clarifies testable transformation pathways for future research and psychological support while avoiding the pathologization, depoliticization, or individualization of realistic climate concern.

## 1 Introduction: eco-anxiety as eco-affective cognition

Environmental change should be approached not only as a background condition for human life, but as a cognitively active field of threat that reorganizes how people perceive, evaluate, imagine, and prepare to act. Psychological responses to climate change involve environmental perception, risk appraisal, uncertainty, emotion, coping, communication, and decision-making (Weissbecker, 2011). Climate risk perception is therefore not reducible to detached analysis: risk judgments are shaped by both analytic evaluation and affective responses at the moment of judgment or decision (Loewenstein et al., 2001; Slovic et al., 2004). Climate change also varies in psychological distance (Trope and Liberman, 2010), and personal experience can make it feel temporally, spatially, or socially closer (McDonald et al., 2015). Empirical work further shows that perceiving climate change as real, human-caused, negative, temporally present, and spatially close is associated with stronger negative affect, higher risk perception, mitigation and adaptation behavior, and policy support (van Valkengoed et al., 2021). Eco-anxiety is consequently a paradigmatic case for studying eco-affective cognition because it condenses environmental threat, future-oriented uncertainty, affect, moral concern, efficacy appraisal, and behavioral readiness into one phenomenon (Clayton and Karazsia, 2020; Contreras et al., 2024; Kurth and Pihkala, 2022).

This framing requires a distinction between legitimate distress and impairing narrowing. Eco-anxiety should not be treated as irrational merely because it is painful: climate-related anxiety is directed toward documented ecological risks and can function as adaptive, future-oriented preparation (Clayton, 2020; Comtesse et al., 2021). The problem emerges when legitimate concern becomes difficult to regulate and reorganizes everyday functioning: climate anxiety measures include cognitive-emotional impairment, rumination-like symptoms, sleep disturbance, concentration problems, and functional impairment in work, family, and social life (Clayton, 2020; Clayton and Karazsia, 2020). Eco-anxiety can thus remain adaptive when it supports reflection and engagement, but may become maladaptive when helplessness, hopelessness, avoidance, or unclear and socially unsupported efficacy pathways reduce action and contribute to eco-paralysis (Comtesse et al., 2021; Heeren et al., 2022; Wullenkord et al., 2021).

The literature is rich but still fragmented. Climate-emotion research now covers anxiety, ecological grief, anger, guilt, shame, hope, hopelessness, overwhelm, empowerment, and related affective states (Pihkala, 2022), while empirical studies show that different eco-emotions have different behavioral correlates: eco-anger has been associated with greater personal and collective pro-climate behavior, whereas eco-anxiety may be less directly mobilizing when other eco-emotions are considered simultaneously (Stanley et al., 2021). Intervention reviews identify cognitive, meaning-focused, emotion-focused, grief-informed, group-based, nature-based, and action-oriented approaches (Baudon and Jachens, 2021; Dailianis, 2020), but these do not yet amount to a concise process model explaining how overwhelming eco-affects are transformed into agency. The missing step is to specify how therapeutic and supportive processes reorganize eco-affective cognition – attention, appraisal, uncertainty, representation, efficacy, and action selection – rather than merely listing symptoms, emotions, or practices.

The present article responds to this fragmentation by developing an Acceptance–Externalization–Agency model of eco-affective cognition. Rather than proposing a new clinical syndrome, ranking therapeutic schools, offering an escape from ecological reality, or claiming that therapy converts anxiety into action, the model asks under which conditions eco-affective distress becomes cognitively and behaviorally narrowing or, alternatively, action-enabling. The model is derived synthetically from converging literatures on climate emotions, affective cognition, acceptance-based processes, symbolic and embodied representation, responsibility appraisal, and efficacy-linked action. The three modalities discussed below were selected because each makes one AEA process especially visible: Morita Therapy foregrounds action-oriented acceptance under persisting affect, Poetry Therapy foregrounds symbolic and linguistic representation, and Psychodrama foregrounds embodied-relational perspective shifting. They are heuristic exemplars, not preferred, exhaustive, or comparatively validated treatments. ACT, CBT, narrative therapy, existential approaches, climate cafés, community psychology, and group-based interventions are not excluded; they may operationalize similar processes in different ways. The argument is therefore theoretical and process-oriented: it builds a conceptual model for future empirical testing rather than conducting a systematic review or claiming efficacy equivalence across modalities (Baudon and Jachens, 2021; de Witte et al., 2021).

## 2 Conceptual clarification: eco-affects, eco-anxiety, and eco-affective cognition

Eco-affects can be defined as a heterogeneous family of affective states that arise in response to ecological disruption, anticipated environmental loss, or socially mediated awareness of climate and biodiversity crises. The term includes threat-related affects such as fear, worry, anxiety, powerlessness, helplessness, and dread; loss-related affects such as sadness, grief, yearning, and solastalgia; moral self- and other-appraisals such as guilt, shame, inadequacy, regret, anger, rage, frustration, betrayal, and moral outrage; and action-oriented or connective affects such as motivation, determination, hope, optimism, empowerment, belonging, togetherness, connection, love, empathy, care, and compassion (Pihkala, 2022). Empirical work likewise treats eco-anxiety, eco-guilt, and eco-grief as multifactorial responses to climate change, environmental trauma, future-oriented distress, coping, and pro-environmental behavior rather than as single intrapsychic symptoms (Ágoston et al., 2022). Eco-affects are therefore environmentally and socially situated: they are evoked by climate change and ecological threat, shaped by contextual factors and collective forms of appraisal, and linked to public-sphere action, personal responsibility, and collective engagement (Bouman et al., 2020; Geiger et al., 2021; Stanley et al., 2021). Conceptually, this situatedness makes eco-affects relational signals about disrupted human–environment relations rather than merely private moods.

Within this broader field, eco-anxiety can be specified as an anticipatory form of eco-affect: it is anxiety, worry, apprehension, or stress significantly related to ecological degradation and anthropogenic climate change, especially to anticipated threats to ecosystems and the perceived collapse of ecological conditions of life (Pihkala, 2020; Wullenkord et al., 2021). Its temporal structure is therefore predominantly future-oriented. General anxiety involves possible threats marked by uncertainty, unpredictability, and limited controllability, and ecological crisis intensifies precisely these conditions because people cannot know how climate impacts will unfold, which losses can still be prevented, or which responses remain within personal or collective control (Dailianis, 2020; Pihkala, 2020). Eco-anxiety is also tied to anticipated environmental loss: degradation, species loss, rising seas, desertification, and threats to ways of life may evoke emotional reactions before the loss has fully occurred, including helplessness, hopelessness, and uncertainty (Comtesse et al., 2021). This definition distinguishes eco-anxiety from a clinical anxiety disorder. It is not classified as an anxiety disorder and should not be pathologized as such, because it can be a rational and adaptive response to real existential threat (Dailianis, 2020; Pihkala, 2020). Clinically relevant eco-anxiety begins where this response becomes severe, persistent, and debilitating – impairing concentration, sleep, relationships, work, family life, leisure, or meaningful climate engagement (Comtesse et al., 2021; Dailianis, 2020; Wullenkord et al., 2021).

On this basis, eco-affective cognition can be defined more narrowly as the dynamic coupling of ecological threat salience, future simulation, responsibility appraisal, and efficacy appraisal under conditions of environmental uncertainty. The concept rejects a simple additive model in which eco-anxiety is an emotion appended to otherwise neutral cognition. Emotion research indicates that affect changes orientation toward stimuli, attentional engagement and disengagement, memory retrieval, appraisals, judgments, and decision-making, with threat-related information being preferentially attended to and anxiety tending to generalize appraisals of risk and uncontrollability across contexts (Bar-Haim et al., 2007; Hayes and Hofmann, 2018). Climate-risk research similarly describes responses to climate change as involving both analytic appraisal and affective, symbolic, and socially mediated sense-making, so that risk communications can make climate change psychologically real as an anticipated, uncertain, and frightening future (Loewenstein et al., 2001; Slovic et al., 2004; Weissbecker, 2011). Eco-affective narrowing, in this stricter sense, occurs when high threat salience, repetitive future simulation, moral over-responsibilization, and unclear or socially unsupported efficacy pathways converge. This moral and practical dimension is central: practical eco-anxiety can sensitize persons to environmentally significant threats and reflect moral attunement to what is ecologically valuable (Kurth and Pihkala, 2022), while different eco-emotions show distinct associations with personal and collective pro-climate behavior (Stanley et al., 2021).

For clarity, four terms organize the argument. Eco-affective cognition denotes the coupling of ecological threat salience, future simulation, responsibility appraisal, and efficacy appraisal under environmental uncertainty. Eco-affective narrowing denotes the constricted condition in which threat dominates attention, future simulation becomes repetitive or catastrophic, responsibility is over-individualized, and credible efficacy pathways recede. Bounded agency denotes the capacity to select and sustain feasible, value-congruent, socially embedded action without assuming control over climate outcomes. Representational externalization denotes the transformation of affective experience into a perceptible, communicable, and revisable form; it does not mean externalizing behavior, denial of responsibility, or displacement of blame.

Agency, in this framework, should therefore not be equated with omnipotent control over ecological outcomes or with optimism that negates loss, uncertainty, or limited personal control. Climate action is a collective-risk domain in which individual action alone cannot solve the problem, and personal helplessness may be structurally intelligible rather than simply demotivating; what matters is whether action is situated within shared capacities, perceived collective efficacy, and attainable roles (Geiger et al., 2021; Wallis and Loy, 2021). Eco-affective agency is thus bounded agency: the capacity to orient attention, appraisal, and behavior toward actions that are possible, communicable, and value-congruent under uncertain conditions. Empirical work links pro-environmental activism to collective efficacy – the belief that an ingroup can achieve environmental protection – and to self-efficacy when personal action is experienced as meaningful within a shared collective context (Wallis and Loy, 2021). Values are also central, because biospheric values and personal or joint responsibility help translate climate worry into mitigation behavior and policy support (Bouman et al., 2020). Hope, accordingly, is not reassurance that the crisis will be solved, but an action-oriented appraisal: hope felt when contemplating public-sphere climate action robustly predicts intentions to act, while “true hope” requires realistic threat appraisal, strategies, support, feedback, and openness to uncertainty (Geiger et al., 2021; Moser and Dilling, 2007).

These distinctions converge on a dynamic criterion: eco-affective cognition is not adaptive or impairing by virtue of negative affect alone, but by how ecological threat is organized in relation to efficacy, responsibility, and meaningful action. Eco-anxiety can function as “practical anxiety” when it sensitizes persons to difficult climate-related decisions and supports reflection and engagement (Kurth and Pihkala, 2022), while worry about climate change may predict support for climate policies and personal mitigation via personal responsibility (Bouman et al., 2020). Conversely, eco-affective cognition becomes vulnerable to impairment when threat appraisal loses its action-orienting structure and persists as debilitating worry, helplessness, avoidance, or the collapse of meaningful engagement (Comtesse et al., 2021; Wullenkord et al., 2021). This distinction prepares the problem model: narrowing begins when ecological threat remains salient while credible pathways for efficacy and meaning recede.

## 3 From environmental threat to cognitive-affective narrowing

Environmental threat enters eco-affective cognition both as direct ecological change and as mediated symbolic information. Direct inputs include experienced or anticipated climate disruption, droughts, heatwaves, ecosystem unpredictability, species disappearance, and pollution, all of which have been reported as elicitors of eco-anxiety, panic, helplessness, or uncertainty among climate-sensitive participants (Ágoston et al., 2022). Broader accounts similarly describe climate and environmental change as already unfolding through increasingly frequent crises that transform human, plant, and animal ecosystems, thereby making environmental degradation a continuous rather than episodic threat context (Desveaux, 2020). These material inputs are amplified by symbolic inputs: documentaries, news articles, mainstream media narratives, and public discourses that present escalating crisis, impending doom, individual responsibility, or insufficient collective help can heighten risk perception and helplessness (Ágoston et al., 2022; Cossman, 2013; Dailianis, 2020). Cognitively, such inputs are likely to become salient when they are appraised as severe, probable, personally or morally relevant, and difficult to control. This logic is consistent with protection-motivation accounts in which perceived threat becomes adaptive only when paired with coping appraisal and self-efficacy (Maddux and Rogers, 1983), and with anxiety models that identify threat appraisal and perceived coping limitations as central to anxiety responses (Emmelkamp and Ehring, 2014). Once appraised in this way, climate-related information can capture attention: meta-analytic evidence indicates that anxious individuals show reliable threat-related attentional bias, and clinical anxiety accounts similarly describe vigilance or hypervigilance toward concern-relevant cues (Bar-Haim et al., 2007; Hofmann et al., 2014; Emmelkamp and Ehring, 2014).

Climate threat is also bodily and spatial before it is fully verbalized. Heat, smoke, drought, flooding, pollution, and the loss of familiar landscapes are not merely informational cues; they may be felt as exhaustion, constriction, vigilance, displacement, or loss of orientation in lived space. Eco-affective cognition therefore includes explicit appraisal, but is not exhausted by propositional thought. Some climate-related affects remain vague, imaginal, preverbal, or difficult to symbolize, especially when the threat is known scientifically while not yet directly seeable, touchable, or locally controllable. This is one reason why symbolic, narrative, artistic, and embodied forms of externalization are theoretically important: they can give form to affective material that has not yet become available as clear verbal cognition (de Witte et al., 2021; Pihkala, 2022).

On this basis, eco-anxiety can be specified as anticipatory cognition: anxiety is future-oriented apprehension about possible threats, closely linked to worry or “apprehensive expectation,” and climate change anxiety extends this process to projected environmental harms even when no direct weather-related event has been personally experienced (Clayton and Karazsia, 2020). This anticipatory structure also fits cognitive-neuroscientific work on prospection, which shows that imagining future events draws on memory-related constructive processes rather than on detached forecasting alone (Schacter et al., 2007). The temporal object of concern is therefore not only present degradation but an uncertain future in which valued places, species, social infrastructures, descendants, and ways of life may be damaged or lost. Qualitative evidence supports this temporal structure: climate-sensitive participants described eco-anxiety as worry for future generations, confusion about ecological unpredictability, and helplessness in response to a challenge perceived as vast and insufficiently controllable (Ágoston et al., 2022). Review work similarly characterizes eco-anxiety as a response to impending climate threats that can include helplessness, hopelessness, uncertainty, cognitive worrying, and apprehension (Comtesse et al., 2021). When this future-oriented cognition becomes habitual, it may shift from constructive problem solving toward repetitive, intrusive, and uncontrollable worry under uncertainty (Verplanken et al., 2020). At its most constricting, eco-anxiety can take the form of catastrophic simulation, in which disastrous futures feel more available than alternative trajectories of mitigation, adaptation, or collective efficacy (Kaplan, 2020; van der Wijngaart, 2021).

The same anticipatory structure is also moralized: climate-related threat is not appraised only as danger, but as an injury involving responsibility, injustice, and violated obligations. Reviews of climate emotions identify guilt/shame, anger/frustration, fear/worry/anxiety, sadness/grief, and hope/empowerment as recurring affective categories, and emphasize that the moral dimension of climate emotions includes disappointment, moral outrage, and desire to act (Pihkala, 2022). Empirical work with young people similarly shows that climate anxiety involves worry, fear, anger, grief, despair, guilt, shame, and hope, while distress is intensified by perceptions that governments and adults have failed to protect ecosystems and future generations (Hickman et al., 2021). In this sense, eco-affective cognition evaluates not only projected environmental loss, but also agency, blame, and legitimacy: the future becomes frightening when valued opportunities are expected to disappear and when institutional inaction is experienced as betrayal, abandonment, injustice, or moral injury (Hickman et al., 2021). These moral emotions can organize motivation in divergent ways. Eco-anger has been associated with greater personal and collective pro-climate behavior, consistent with the idea that perceived unfairness can mobilize action (Stanley et al., 2021). Yet moralization can also narrow cognition when responsibility is individualized: environmental discourse may shift responsibility onto consumers and families, making anxiety a self-governing burden (Cossman, 2013). When perceived agency is low, guilt, shame, powerlessness, and helplessness may therefore amplify self-blame rather than support effective engagement (Hickman et al., 2021; Stanley et al., 2021).

Efficacy appraisal is therefore the hinge at which eco-anxiety becomes either mobilizing concern or impairing cognitive-affective narrowing, but this hinge should not be understood as a simple linear effect of “low efficacy.” The relevant determinant is whether climate-related affect can be linked to specific, socially embedded, and subjectively meaningful pathways for action. Emotional reactions influence decision-making differently depending on whether they arise in relation to possible actions and whether those actions appear capable of meeting valued goals (Geiger et al., 2021; Lerner et al., 2015). In survey research on public-sphere climate action, hope when contemplating action was associated with climate-action intentions, whereas anxiety and helplessness showed weaker associations, suggesting that affect becomes behaviorally consequential when linked to an actionable pathway rather than to threat exposure alone (Geiger et al., 2021). Similarly, worry about climate change may translate into action when it is connected to responsibility and possible joint efforts, while efficacy beliefs are identified as a likely pathway through which values and worry become climate action (Bouman et al., 2020). Collective efficacy is especially central: in the context of pro-environmental activism, it refers to the belief that an ingroup can jointly achieve environmental protection, and prior studies associate it with protest intentions and climate-protective activism (Wallis and Loy, 2021). Conversely, when no credible individual or collective route is appraised as available, concern can become narrowed into helplessness, non-participation, or withdrawal (Stanley et al., 2021).

The central problem is therefore not negative climate affect as such, but the loss of an action-orienting structure. Eco-affective narrowing occurs when ecological threat remains salient while future simulation becomes repetitive, responsibility becomes over-individualized, and efficacy pathways are unclear, unavailable, or socially unsupported. Clinical relevance should be inferred not from the presence of climate concern, but from persistence, uncontrollability, functional impairment, avoidance, and the collapse of meaningful engagement. The AEA model addresses this transition point by asking how legitimate distress can be accepted, represented, contextualized, and linked again to bounded forms of agency.

## 4 The acceptance–externalization–agency model of eco-affective cognition

The Acceptance–Externalization–Agency (AEA) model (Figure 1) is proposed as a cognitive-affective process model linking environmental input to bounded agency without reducing eco-anxiety to psychopathology. Its three operations are not inferred as a validated causal sequence, but selected as conceptual nodes that correspond to three recurrent points at which eco-affective cognition may narrow: secondary struggle against affect, immersive or insufficiently symbolized distress, and the collapse of credible efficacy pathways. Environmental inputs include lived ecological disruption, visible degradation, biodiversity loss, pollution, mediated reports, political inaction, and future-oriented uncertainty. These inputs are filtered by contextual conditions, including direct exposure to ecological disruption, socioeconomic vulnerability, institutional trust or betrayal, cultural and place-based meaning, social recognition or isolation, and access to collective action. They then enter eco-affective appraisal through threat, uncertainty, loss, empathy, responsibility, injustice, controllability, and self- or collective efficacy. When threat remains salient while specific, socially embedded, and subjectively meaningful pathways for response recede, eco-affective cognition may narrow into attentional capture, repetitive future simulation, moral over-responsibilization, avoidance, and action-selection collapse (Clayton and Karazsia, 2020; Comtesse et al., 2021; Hickman et al., 2021; Wullenkord et al., 2021).

**Figure 1:**
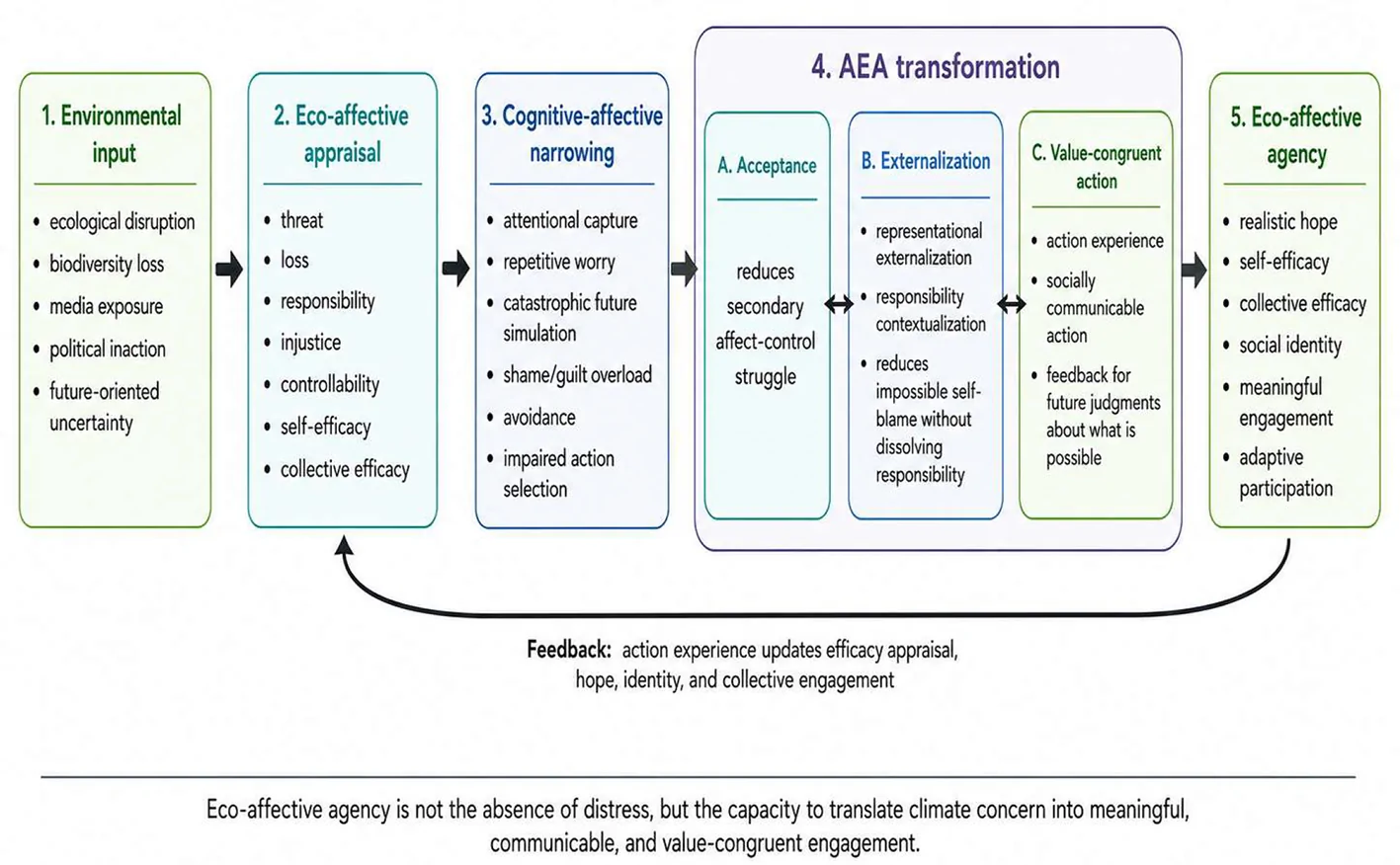
The Acceptance–Externalization–Agency (AEA) model of eco–affective cognition.

The transformative layer of the model specifies three linked operations rather than a deterministic mechanism. First, acceptance reduces secondary affect-control struggle: climate emotions are treated as legitimate internal events that can be carried while acting, rather than as symptoms that must be eliminated before action becomes possible. Second, externalization changes the representational and social status of eco-affect. Here the model distinguishes representational externalization, in which diffuse affect becomes nameable, writable, metaphorical, scenic, embodied, and observable, from responsibility contextualization, in which distress is situated within political, ecological, and collective conditions rather than individualized as private failure. This distinction is crucial: contextualizing responsibility should reduce over-individualized self-blame, but agency is weakened if personal, collective, and institutional responsibility disappear altogether. Third, value-congruent action changes efficacy appraisal through experience: action becomes autobiographical, socially communicable, and available as feedback for future judgments about what is possible (Bouman et al., 2020; Geiger et al., 2021; Wallis and Loy, 2021).

Although the model is depicted sequentially for analytic clarity, the process should be read as recursive rather than strictly linear. Acceptance and externalization may need to be revisited when new climate information, bodily exposure, institutional inaction, or personal loss reactivates distress. AEA is therefore not a promise that representation automatically produces agency. It proposes that supportive contexts can help persons move back and forth among tolerating affect, giving it form, contextualizing responsibility, selecting feasible action, and using action experience as feedback for future appraisals of threat, hope, identity, and efficacy.

In practical terms, the model can be used as an orienting framework in psychotherapy, psychosocial counseling, climate education, climate cafés, group reflection, and activist or community settings. It does not prescribe one intervention. Rather, it asks four practical questions: which climate-related affect is being struggled against? How can this affect be represented or shared? At which scale does responsibility belong—personal, collective, institutional, or some combination of these? Which bounded action is sufficiently feasible, value-congruent, and socially supported to generate feedback for agency? In this sense, AEA is intended to preserve rather than weaken contact with ecological reality.

## 5 Relation to existing models and incremental contribution

The AEA model overlaps with several established frameworks but is not reducible to any one of them (Table 1). It shares with protection-motivation and coping-appraisal approaches the idea that threat alone does not produce adaptive action unless perceived coping options and efficacy are available (Maddux and Rogers, 1983); in the present account, narrowing is most likely when high threat salience is not linked to specific, socially embedded, and subjectively meaningful action pathways. It also overlaps with ACT and acceptance-based approaches in treating distress as something that can be carried while acting in accordance with values rather than eliminated before action begins (Hayes and Hofmann, 2018). It converges with meaning-focused climate coping and constructive hope by distinguishing realistic, action-oriented hope from denial-based reassurance (Ojala, 2012; Pihkala, 2022). Finally, it aligns with social-identity and collective-efficacy accounts by treating agency as socially embedded rather than purely intrapsychic (Barth et al., 2021; Bouman et al., 2020; Fritsche et al., 2018; Geiger et al., 2021; Wallis and Loy, 2021).

**Table 1:** Relation of the AEA model to selected existing frameworks.

Framework	Main focus	Limitation for the present argument	Specific contribution of the AEA model
Protection motivation theory	Threat appraisal, coping appraisal, self-efficacy	Remains a general risk model	Adds representational externalization and responsibility contextualization
ACT and acceptance-based approaches	Acceptance, values, committed action	Not specific to ecological threat, political responsibility, or collective agency	Links acceptance to climate-specific moral appraisal, collective efficacy, and bounded agency
Meaning-focused coping and constructive hope	Meaning, hope, positive reappraisal	Provides less detailed process logic of representation	Describes externalization as a transition from affective immersion to action clarity
SIMPEA and collective-efficacy models	Social identity, collective efficacy, collective action	Focuses less on internal affect organization	Connects collective efficacy with acceptance and externalization
Narrative and arts-based approaches	Externalization, symbolic distancing, symbolic transformation	Often leaves the efficacy feedback loop implicit	Links symbolic representation to value-congruent action and subsequent efficacy updating

The incremental contribution of the AEA model is explicit in four respects. First, it conceptualizes eco-anxiety as an eco-affective cognitive configuration rather than only as an emotion, symptom, coping style, or behavioral predictor. Second, it integrates acceptance, externalization, responsibility contextualization, and efficacy-linked action within one recursive transformation process. Third, it gives externalization a specific role as the conversion of immersive, sometimes preverbal or embodied affect into symbolic, narrative, relational, or scenic form. Fourth, it defines agency as bounded, value-congruent, and socially embedded rather than as individual control over ecological outcomes. This integration is especially important for climate-related cognition because ecological threat is not merely a stimulus to be appraised, but a morally and socially mediated future that can become immersive, self-blaming, and paralyzing. The model therefore explains how threat salience and uncertainty may become narrowing, and how symbolic, relational, and action-based processes may reorganize attention, future simulation, responsibility, efficacy, and decision-making.

The model's responsibility component distinguishes personal, collective, and institutional levels. Personal responsibility refers to feasible practices such as communication, participation, consumption choices, professional conduct, or value-congruent everyday decisions. Collective responsibility refers to coordinated group capacities, social movements, professional communities, and shared norms. Institutional responsibility refers to political, economic, legal, and infrastructural actors with disproportionate power to shape mitigation, adaptation, and exposure. Reducing excessive self-blame therefore does not mean dissolving responsibility; it means locating responsibility at the appropriate scale while preserving the possibility of meaningful participation.

## 6 Processes of transformation: conceptual exemplars

### 6.1 Acceptance as attentional reorientation

Acceptance denotes an active reorganization of the person's relation to affect rather than passive resignation. In Morita therapy, anxiety and other emotions are treated as naturally fluctuating experiences that need not be denied, suppressed, or manipulated before life can proceed; attention is redirected from symptom fixation toward objective observation, constructive behavior, and socially meaningful activity (Reynolds, 1989; Corsini, 2001). The same process appears in acceptance-oriented approaches more broadly: suffering is maintained not only by distress itself, but by narrowed attention, judgment, reactivity, and rigid attempts to avoid or control internal experiences; acceptance and mindfulness alter this relationship and support meaningful engagement rather than symptom elimination (David et al., 2018; Emmelkamp and Ehring, 2014). For eco-anxiety, acceptance therefore interrupts the recursive capture of attention by anxiety about anxiety. Climate concern does not have to disappear before action can begin; purposeful, value-congruent behavior can occur with fear present, and relief, when it occurs, is a by-product of reduced emotional struggle and renewed practical involvement rather than the condition of agency (Reynolds, 1989).

Morita therapy makes this shift concrete under conditions of uncertainty. Reynolds describes treatment as moving attention away from symptoms and toward constructive behavior, so that clients learn to make intelligent and responsible choices despite moods, uncertainty, or discomfort (Reynolds, 1989). This is crucial for eco-anxiety because climate threat rarely permits complete reassurance: scientific, political, and personal uncertainties cannot be resolved before action is required. The Waltraud vignette refers to a previously published illustrative case narrative in Raile (2023a). It is used here not as a documented outcome case or as evidence for Morita Therapy, but as a conceptual example of how climate-related fear may remain affectively present while outward action is reorganized. The vignette illustrates the AEA distinction between inner affective experience and value-congruent action under uncertainty. The relevant movement is not from anxiety to emotional calm, but from overwhelming worry to concrete, value-congruent decisions. The case thus illustrates a theoretical process; it does not establish causal efficacy.

### 6.2 Externalization as representational distancing

Externalization should be understood first as a representational operation. It does not denote externalizing behavior, displacement of responsibility, or the evacuation of affect into the outside world. This distinction is important because externalizing and internalizing have a separate history as broad dimensions of behavioral and emotional problems in developmental psychopathology (Achenbach and Edelbrock, 1978). In AEA, by contrast, externalization refers to a symbolic, narrative, or embodied operation: writing, metaphor, narrative, art, or enactment can convert diffuse eco-affects into perceptible forms that can be named, placed, revised, and addressed. This use overlaps with, but is not identical to, adjacent processes. Emotional labeling names affect; cognitive defusion changes the person's relation to thoughts; narrative distancing creates reflective distance through story; and perspective-taking shifts the social or imaginative position from which distress is viewed. AEA externalization may include these operations, but specifies their climate-affective function more narrowly: immersive distress is converted into a communicable and revisable representation that can support responsibility calibration and subsequent action clarity. Poetry therapy provides a useful exemplar because the RES model describes three interrelated modes: receptive/prescriptive work with existing texts, expressive/creative writing, and symbolic/ceremonial practices that organize experience through image, ritual, and meaning (Mazza and Hayton, 2013). Jaeger similarly defines poetry therapy through language, symbol, and story, including awareness of cognitive and emotional states, meaning-making, connection to self and others, and transcription of ideas and emotions into written form (Jaeger, 2023). In eco-affective terms, metaphor and writing create transitional distance: anxiety becomes observable, re-readable, shareable, and open to narrative reconfiguration rather than remaining an undifferentiated affective pressure.

The transformative potential of externalization lies precisely in combining, rather than separating, internal and external dimensions. A poem, image, metaphor, scene, or bodily posture is neither simply inside the person nor simply outside; it becomes a mediating form through which affect, ecological reality, and social communication can be jointly apprehended. This use is close to narrative therapy, where externalizing conversations separate persons from problem-saturated self-descriptions without denying their lived relation to the problem (White and Epston, 1990). Externalizing problems through art and journaling has likewise been described as supporting emotional expression, resource recognition, agency, and empowerment (Keeling and Bermúdez, 2006). For eco-anxiety, however, externalizing responsibility is theoretically delicate. Climate distress should not be individualized as personal pathology, yet personal responsibility can mediate the relation between climate worry and mitigation behavior (Bouman et al., 2020). The AEA model therefore treats contextualization as agency-supportive only when it reduces shame and impossible self-blame while preserving feasible personal, communicative, collective, and institutional responsibility. Externalization should create distance from immobilizing self-identification with the problem, not distance from ecological concern itself.

This distinction also clarifies the link between externalization and hope. Raile's discussion of eco-anxiety in poetry therapy treats writing about climate anxiety as a way of reflecting on thoughts and feelings and introduces adapted therapeutic-writing exercises illustrated through semi-fictional case texts (Raile, 2023a). The decisive change is not certainty that ecological outcomes will be benign, but the creation of symbolic room for a next possible act. Climate-emotion research separates hopeful agency from mere optimism: Pihkala notes that “gritty” or “radical” hope may not be strongly tied to optimism, while Ojala's distinction between constructive hope and denial-based hope is important for climate contexts (Ojala, 2012; Pihkala, 2022). Empirically, hope felt when contemplating one's own public-sphere climate action robustly predicted intentions to act, whereas anxiety and helplessness did not (Geiger et al., 2021). Externalization therefore supports agency when it reorganizes future simulation: uncertainty remains, but it is no longer encoded only as impossibility.

### 6.3 Perspective shifting and social-cognitive agency

Psychodrama provides a conceptual exemplar of embodied and relational externalization. Rather than treating eco-anxiety only as reportable content, psychodramatic techniques make affect available as spatial arrangement, bodily action, interpersonal position, and group response. Scene-setting, concretization, mirroring, doubling, auxiliary egos, and role reversal can represent abstract internal dynamics, loosen rigid narratives, and enable alternative responses in an emotionally safe space (Fávero et al., 2025). Applied to eco-anxiety, the fear can be staged as “the news,” “the fear,” a future person, an affected community, a policymaker, or a non-human actor, allowing the protagonist to perceive climate distress as an addressable relational configuration rather than an undifferentiated private burden (Raile, 2023b).

The central cognitive operation here is perspective shifting. Role reversal invites the person to adopt another position and has been associated with empathy, perspective broadening, decentering, self-awareness, conflict resolution, and acceptance of others' experiences (Fávero et al., 2025). For eco-affective cognition, this matters because climate distress is organized around perceived relations among self, others, institutions, non-human nature, and future generations, not only around private fear. Psychodramatic mirroring and role reversal may therefore support agency by making the social field of responsibility visible: the protagonist can experience being witnessed, imaginatively occupy positions of others, and test value-congruent responses within a group. This fits intervention reviews emphasizing social connection, group belonging, emotional support, and meaningful individual or collective action as recurrent resources for eco-anxiety (Baudon and Jachens, 2021; Dailianis, 2020).

## 7 Conceptual propositions

Proposition 1: Eco-affective narrowing is most likely when high ecological threat salience and uncertainty are not linked to specific, socially embedded, and subjectively meaningful pathways for action. Eco-anxiety is not impairing simply because environmental threat is perceived; it becomes clinically relevant when worry is persistent, difficult to control, functionally impairing, avoidant, or disconnected from meaningful engagement (Clayton, 2020; Clayton and Karazsia, 2020; Wullenkord et al., 2021). This proposition can be tested by modeling interactions among threat appraisal, repetitive climate-related worry, functional impairment, perceived response efficacy, collective efficacy, and availability of concrete action pathways.

Proposition 2: Acceptance reduces secondary affect-control struggle and preserves attentional flexibility. Attempts to eliminate legitimate climate distress may intensify symptom fixation when the underlying threat is real and morally meaningful (Clayton, 2020; Kurth and Pihkala, 2022). Acceptance-oriented interventions are therefore expected to reduce climate-related experiential avoidance, symptom monitoring, and secondary affect-control struggle without necessarily reducing climate concern itself; outcomes should include rumination, willingness to remain engaged with climate information, and value-congruent action readiness.

Proposition 3: Representational externalization converts immersive eco-affect into observable, shareable, and revisable form. Writing, metaphor, art, narrative, and enactment are hypothesized to reduce affective immersion when they help persons name, locate, symbolize, and communicate distress rather than remain fused with it (Keeling and Bermúdez, 2006; Mazza and Hayton, 2013; White and Epston, 1990). Empirically, representational externalization should increase emotional differentiation, narrative coherence, perspective taking, and perceived action clarity rather than simply reducing negative affect. This proposition would be weakened if externalization produced only expressive release without changes in action clarity, responsibility calibration, or efficacy appraisal; if it increased rumination, catastrophic imagery, avoidance, or responsibility diffusion; or if its effects disappeared once collective efficacy and social support were taken into account. Responsibility contextualization should be distinguished from this representational process: it is agency-supportive when it reduces impossible self-blame while maintaining feasible personal, collective, and institutional responsibility.

Proposition 4: Value-congruent action updates efficacy appraisal through action experience and social feedback. Hope should not be conceptualized as denial of ecological risk, but as an affective-cognitive orientation toward possible action under uncertainty. Because hope felt when contemplating public-sphere climate action was associated with intentions to act more robustly than anxiety or helplessness, action experience should alter subsequent appraisals of what is possible (Geiger et al., 2021). Testable outcomes include action-specific hope, self-efficacy, collective efficacy, and perceived response efficacy before and after value-congruent action, especially when the action is socially recognized or embedded in collective identity.

Proposition 5: Eco-affective agency is sustained when individual action is embedded in collective efficacy, moral meaning, and social identity. Because climate change cannot be solved by isolated individuals, agency is most adaptive when personal regulation is linked to communicative, identity-based, and collectively efficacious forms of action (Barth et al., 2021; Fritsche et al., 2018; Wallis and Loy, 2021). This proposition can be tested with outcomes that distinguish individual behavioral intention, collective-action intention, social identification, perceived collective efficacy, functional impairment, and meaningful engagement. It also limits the model ethically: therapeutic success should not mean depoliticizing climate concern but reducing paralysis while preserving the moral and political intelligibility of ecological distress.

## 8 Research and support implications

For research, the AEA model translates eco-affective agency into tractable questions about how climate threat is selected, represented, and transformed. Experimental writing tasks could test whether externalization changes attentional engagement with climate information, reduces repetitive climate-related worry, or shifts catastrophic anticipation toward value-congruent action. Climate anxiety measures already distinguish cognitive-emotional impairment, functional impairment, behavioral engagement, and perceived efficacy, making such shifts empirically measurable (Clayton and Karazsia, 2020). A second research line should examine perspective shifting as an embodied or imaginative manipulation of social cognition: role reversal with future persons, affected communities, policymakers, or non-human actors could be tested for effects on perceived self-efficacy, collective efficacy, and pro-environmental decision-making. A third line should examine media exposure, doomscrolling-like repeated threat input, and future imagery, because continuous negative climate information can increase anxiety without automatically translating into pro-environmental intention (Kapeller and Jäger, 2020; Kaplan, 2020; Verplanken et al., 2020).

Intervention and process studies could directly test whether acceptance changes the transition from eco-affective distress to action readiness. Participants with elevated climate anxiety could be randomized to an acceptance-oriented protocol – validation, emotional processing, self-care, and meaning-focused clarification – or to structurally equivalent psychoeducation, with outcomes including repetitive worry, cognitive-emotional impairment, willingness to remain engaged with climate information, and intention to act (Baudon and Jachens, 2021). Multi-method designs would be particularly useful: EMA-style diary studies can model climate emotions as unfolding processes (Contreras et al., 2024); writing and decision tasks can test representational externalization; and scale-based models should include climate anxiety, rumination, hope, self-efficacy, collective efficacy, and pro-environmental behavior (Geiger et al., 2021; Wullenkord et al., 2021).

For psychological support, the model implies a stance of validation without therapeutic passivity. Climate-related fear, grief, anger, and helplessness are not in themselves evidence of irrationality, but they may become clinically relevant when they narrow attention, emotion regulation, relationships, sleep, work, study, or action (Clayton and Karazsia, 2020; Cianconi et al., 2023). Support should therefore neither reassure clients away from the climate crisis nor intensify catastrophic preoccupation. It should legitimate the concern, assess impairment, and cultivate three process dimensions: acceptance of distress without making action conditional on emotional comfort; symbolic or embodied externalization that makes diffuse affect shareable and reflective; and bounded agency that connects values to feasible individual, communicative, and collective forms of action (Baudon and Jachens, 2021; Budziszewska and Jonsson, 2021).

Eco-affective agency should also be framed as socially embedded rather than as a purely intrapsychic coping achievement. Climate-related anxiety can be absorbed into a broader politics of individual responsibility, in which people are urged to manage environmental risk through private consumption and self-governance while structural causes remain displaced onto personal lifestyle correction (Cossman, 2013). Psychological support should therefore avoid converting ecological distress into another individualized self-regulation task. Instead, it can help persons distinguish what is personally actionable from what requires communication, shared norms, social identity, and collective coordination. Individual coping becomes more adaptive when it is connected to values-based communication and collective efficacy rather than confined to private symptom management (Barth et al., 2021; Wallis and Loy, 2021).

## 9 Limitations and conclusion

Several limitations follow from the article's theoretical design. The argument is a conceptual analysis, not a systematic review, scoping review, or empirical outcome study; it does not claim exhaustive search coverage, formal inclusion criteria, or quantitative synthesis. The clinical material and previously published case-based discussions are used illustratively: they can convey therapeutic process, but they are vulnerable to selection, memory, and confirmatory biases and cannot substitute for recorded, experimental, or controlled evidence (Messer and Gurman, 2011). This caution is especially important because eco-anxiety intervention literature remains heterogeneous and empirically limited (Baudon and Jachens, 2021). Morita Therapy, Poetry Therapy, and Psychodrama are therefore not proposed as superior or representative treatments. They function as conceptual exemplars of acceptance, representational externalization, perspective shifting, and efficacy-linked action. The AEA model should accordingly be treated as hypothesis-generating: its process assumptions require experimental, longitudinal, qualitative, and mixed-method evaluation, and externalization should not be assumed to be universally beneficial across persons, contexts, or forms of climate distress.

A further ethical and political limitation is that the proposed processes must not be read as relocating responsibility for climate distress onto isolated individuals. Climate-related distress arises in relation to real and accelerating environmental changes, unequal exposure, and political-economic power (Clayton, 2020; Hayes et al., 2018). Psychological work may help persons and communities tolerate affect, sustain agency, and make meaning, but it cannot substitute for mitigation, adaptation, institutional coordination, and collective mobilization. The point is not to diminish clinical care, but to delimit it: support should reduce paralysis and shame while preserving the moral and political intelligibility of climate concern.

Finally, the model explicitly includes unconscious, preverbal, and embodied dimensions of eco-affect as inputs to representation rather than as marginal additions. A cognitive-affective model can describe how threat salience, future simulation, responsibility, and efficacy are organized, but it should not imply that climate-related uneasiness is always fully conscious, verbal, or conceptually available. Some eco-affects may be experienced as bodily tension, wordless dread, vague anticipatory unease, ecological grief, or environmental melancholia before they can be clearly stated (Cunsolo and Ellis, 2018; Lertzman, 2015). The arts-based and embodied examples are therefore included because they may help such affective material become symbolically and socially available, even though the present article does not offer a full psychoanalytic, phenomenological, or arts-therapy theory of these dimensions.

In conclusion, eco-anxiety is best understood not as a discrete symptom to be eliminated, but as an eco-affective cognitive configuration through which environmental threat reorganizes attention, future simulation, responsibility appraisal, efficacy judgment, and action selection. The AEA model contributes a concise framework for this process. Acceptance reduces secondary struggle against legitimate distress; externalization transforms immersive eco-affect into representational and relational distance; responsibility contextualization reduces impossible self-blame without dissolving personal and collective responsibility; and value-congruent action updates efficacy through experience and social feedback. Eco-affective agency names the resulting stance: perceiving danger, interpreting its meaning, and acting responsibly without requiring certainty, emotional comfort, or the disappearance of anxiety.
